# Predictive Factors of Lymph Node Metastasis in Patients With Papillary Microcarcinoma of the Thyroid: Retrospective Analysis on 293 Cases

**DOI:** 10.3389/fendo.2020.00551

**Published:** 2020-08-25

**Authors:** Fabio Medas, Gian Luigi Canu, Federico Cappellacci, Francesco Boi, Maria Letizia Lai, Enrico Erdas, Pietro Giorgio Calò

**Affiliations:** ^1^Department of Surgical Sciences, University of Cagliari, Cagliari, Italy; ^2^Endocrinology, Department of Medical Sciences and Public Health, University of Cagliari, Cagliari, Italy; ^3^Division of Anatomy and Pathological Histology, University of Cagliari, Cagliari, Italy

**Keywords:** thyroid carcinoma, lymph node metastasis, microcarcinoma, thyroidectomy, lymph node dissection

## Abstract

**Introduction:** Papillary thyroid microcarcinoma (PTMC) is defined as a tumor with a larger diameter ≤ 1 cm and is considered having an indolent course and an excellent prognosis. Nevertheless, the incidence of lymph node metastasis in PTMC is not negligible, reaching up to 65% in some series. The aim of this study was to assess the incidence of lymph node metastasis in patients with PTMC and to evaluate predictive factors for lymph node metastasis.

**Methods:** We included in this retrospective observational study patients who underwent thyroidectomy with pathological diagnosis of PTMC at our department from January 2003 to June 2019.

**Results:** Two hundred ninety-three patients were included in the study. The incidence of lymph node metastasis was 13.7%. Multivariate analysis revealed as independent risk factors for lymph node metastasis age <45 years, nodule size ≥6 mm, tall cell variant of PTC, extrathyroidal extension, and angioinvasion. Conversely, autoimmune thyroiditis was found as a protective factor for lymph node metastasis. A subgroup of patients, with nodule size ≤ 5 mm, presented non-aggressive features.

**Conclusion:** The incidence of lymph node metastasis in PTMC is considerable; the size of the tumor appears to be the most significant predictive factor for lymph node metastasis. The traditional cut-off value used for definition of microcarcinoma could be reconsidered to identify patients with an indolent course of the tumor, where active surveillance could be the appropriate treatment, and on the other hand, patients with potentially aggressive tumors requiring an adequate surgical intervention.

**Clinical Trial Registration:** The trial was registered at ClinicalTrials.gov (ID: NCT04274829).

## Introduction

Papillary thyroid carcinoma (PTC) is the most common variety of differentiated thyroid carcinoma. Its incidence has increased in the last decades, mainly due to the diffusion of ultrasound (US) examination of the neck (1–3). Furthermore, the diffusion of screening programs for thyroid cancer has allowed detecting a larger number of tumors at an early stage. Papillary thyroid microcarcinoma (PTMC) is defined as a tumor with the larger diameter ≤ 1 cm (4, 5). In the absence of aggressive features, the American Thyroid Association (ATA) guidelines include this entity in the low-risk category, with a risk of recurrence estimated as 1–2% (6); on the other side, if aggressive characteristics are present, the risk of relapse is not negligible, with lymph node (LN) metastases and extrathyroidal extension being the most important predictive factors of recurrence. Because of its indolent course and its excellent prognosis, in the last few years, active surveillance has been purposed as an alternative to immediate surgery (7). Nevertheless, the incidence of lymph node metastasis in PTMC is not negligible, also in patients with clinically uninvolved lymph nodes (cN0), reaching approximately up to 65% in some series (8–11); this finding raises questions on the most appropriate treatment for PTMC.

The aim of this study was to assess the incidence of lymph node metastasis in patients with PTMC and to evaluate predictive factors for lymph node metastasis.

## Methods

We included in this retrospective observational study patients who underwent thyroidectomy at our department from January 2003 to June 2019 with pathological diagnosis of PTMC, defined as a PTC with a larger diameter equal or inferior to 1 cm. Patients were selected from a prospectively institutional maintained database including all patients with thyroid carcinoma. Exclusion criteria were concomitant tumors with a larger diameter >1 cm, patients in which an evaluation of lymph node status was not available because any sample of lymph node was obtained during surgery, and those with incomplete data.

The primary outcome of the study was the incidence of lymph node metastasis in PTMC, and the secondary was to identify independent risk factors for lymph node metastasis.

Preoperative assessment included anamnesis, physical examination, blood tests to assess thyroid function and autoimmune thyroiditis, and high-resolution US of the neck with careful evaluation of the central and of the lateral compartment of the neck. Fine needle cytology aspiration (FNAC) was performed in case of suspicious nodules; the cut-off value for FNAC was 5 mm. An FNAC with dosage of Thyroglobulin (Tg) was performed in case of positive lymph nodes at US in central or lateral neck compartment. Thyroid scintigraphy was done only in case of hyperthyroidism, defined as low serum TSH (<0.4 mIU/L) and normal or high serum FT3 and FT4. Preoperative laryngoscopy was routinely performed to assess vocal fold mobility.

Surgical procedure consisted of extracapsular total thyroidectomy (TT) or lobo-isthmectomy (LH). In case of low-risk tumors with clinically uninvolved lymph nodes (cN0), a lymph node sampling of the central compartment was performed. Prophylactic central lymph node dissection (CLND) was done in case of clinically uninvolved central lymph neck nodes (cN0) in high-risk tumors, including patients with preoperative US or intraoperative suspicion of extracapsular extension of the tumor, or in case of clinically involved lateral neck nodes (cN1b), as suggested from ATA guidelines. A therapeutic CLND was performed in case of preoperative or intraoperative suspicion of lymph node metastasis of the central compartment. Therapeutic lateral neck dissection (LND) was performed only in case of preoperative evidence of lateral neck node metastasis.

In case of multifocal tumor, the tumor size was determined as the larger diameter of primary tumor. Extrathyroidal extension was defined as the presence of gross infiltration of perithyroidal tissues found at pathological examination, and vascular invasion as the invasion of vessels in the tumor capsule or beyond, with intravascular tumor cells attached to the vessel wall.

Autoimmune thyroiditis was defined in case of positive Thyroglobulin-Antibodies (Tg-Ab) and/or Thyroid Peroxidase-Antibodies (TPO-Ab) and confirmed at pathological examination.

Tall cell variant of PTC was defined in case of presence of columnar cells (height is twice the width) representing at least 50% of tumor cells.

Lymph node yield was defined as the number of lymph nodes retrieved after lymphectomy, and lymph node ratio as the ratio of metastatic lymph nodes out of the total lymph nodes removed.

Four preoperative and seven pathologic features were tested as risk factors for lymph node metastasis: sex, age, hyperthyroidism, autoimmune thyroiditis, size of the tumor, thyroid weight, histological subtype of PTC, multicentricity, angioinvasion, and the presence of extrathyroidal extension.

Univariate analysis was conducted using chi-squared test for categorical variables and Student's *t*-test for continuous variables. Factors with a *p*-value ≤ 0.10 in univariate analysis were considered potentially significant and were included in the multivariate analysis. Logistic regression was employed to identify independent risk factors for lymph node metastasis; results were considered statistically significant for *p*-value < 0.05.

Calculations were performed with MedCalc® vers. 19.1.3. Continuous variables are reported as the mean ± standard deviation of the mean.

## Results

During the study period, a total number of 584 patients with pathological diagnosis of PTMC underwent surgical therapy; 240 patients were excluded because pathologic N status was not assessed, and 51 because of incomplete data. Therefore, 293 patients met inclusion criteria and were included in the study.

Preoperative data are reported in Table 1. Diagnosis of PTMC was incidental in 115 (39.2%) patients, whereas it was suspected at preoperative exams in 178 (60.8%) cases. There were 61 (20.8%) males and 232 (79.2%) females, with a mean age of 49.8 ± 14.3 years. Autoimmune thyroiditis was present in 175 (59.7%) cases, and 44 (15%) patients had a hyperthyroidism status (21 had a Graves' disease, 21 a toxic multinodular goiter, and 3 a toxic adenoma).

**Table 1:** Preoperative data and surgical procedure.

	**Patients (*n* = 293)**
**Sex**	
Male	61 (20.8%)
Female	232 (79.2%)
Age, years (range)	49.8 ± 14.3 (15–80)
Hyperthyroidism	44 (15%)
Autoimmune thyroiditis	175 (59.7%)
Preoperative diagnosis of PTMC	178 (60.8%)
- Hypoechoic nodule	121 (67.9%)^*^
- Microcalcifications	21 (11.8%)^*^
- Intranodular vascularization	84 (47.2%)^*^
Preoperative diagnosis of metastatic lymph nodes at US	11 (3.8%)
**Surgical procedure**	
- LH + LNS	9 (3.1%)
- LH + CLND	2 (0.7%)
- TT + LNS	175 (59.7%)
- TT + CLND	100 (34.1%)
- TT + CLND + LND	7 (2.4%)

US, ultrasound; TT, total thyroidectomy; LH, lobo-isthmectomy; LNS, lymph node sampling; CLND, central compartment lymph node dissection; LND, lateral neck dissection.

* *Calculated on 178 patients with preoperative diagnosis of PTMC*.

Among the 178 patients with a preoperative suspicion of PTMC, US examination demonstrated a hypoechogenic nodule in 121 (67.9%) cases, microcalcifications in 21 (11.8%) patients, and intranodular vascularization in 84 (47.2%) cases.

A lobo-isthmectomy associated with lymph node sampling of the central compartment was performed in 9 (3.1%) cases and with complete CLND in 2 (0.7%) patients. Total thyroidectomy with lymph node sampling was performed in 175 (59.7%) cases, associated with prophylactic or therapeutic CLND in 100 (34.1%) patients and to LND in 7 (2.4%) cases.

Full pathological features are reported in Table 2. Overall, the lymph node yield was 4.3 ± 4.8; specifically, the lymph node yield was 2.1 ± 1.1 in lymph node sampling, 7.3 ± 4.7 in CLND, and 24.8 ± 8.16 in LND.

**Table 2:** Pathological features of 293 patients with papillary thyroid carcinoma.

	**Patients (*n* = 293)**
Nodule size, mm (range)	5.8 ± 2.9 (0.5–10)
Thyroid weight, g (range)	32.1 ± 26.7 (8–164)
**Histotype**	
PTC	182 (62.1%)
FV-PTC	63 (21.5%)
Tall cell carcinoma	48 (16.4%)
Extrathyroidal extension	23 (7.8%)
Multicentric carcinoma	97 (33.1%)
Angioinvasive carcinoma	19 (6.5%)
Lymph node yield (range)	4.3 ± 4.8 (1–33)
Lymph node size, mm (range)	0.7 ± 0.4 (0.4–21)
Lymph node metastasis	40 (13.7%)
Unexpected lymph node metastasis^*^	29 (9.8%)
Number of involved LN per patient^**^ (range)	2.5 ± 2.4 (1–12)
Lymph node ratio^**^ (range)	0.49 ± 0.32 (0.06–1)
Extranodal extension	4 (1.4%)

PTC, papillary thyroid carcinoma; FV-PTC, follicular variant of PTC.

* Pathological diagnosis of lymph node metastasis that was unsuspected at preoperative US examination.

** *Calculated on 40 patients with lymph node metastasis*.

The incidence of lymph node metastasis was 13.7%, with a lymph node ratio of 0.49 ± 0.32. Specifically, the incidence of metastasis of the central compartment was 12.9%, and that of the lateral neck compartment was 2%. Unexpected lymph node metastasis was found in 29 (9.8%) patients, whereas in 11 (3.8%) patients, pathological examination confirmed preoperative US finding. Extranodal extension was found in 4 (1.4%) patients.

During univariate analysis, age, autoimmune thyroiditis, nodule size, tumor histotype, extrathyroidal extension, and angioinvasion were found as potentially significant risk factors for lymph node metastasis (Table 3).

**Table 3:** Univariate and multivariate analyses of preoperative data and pathological features of 293 patients with papillary thyroid microcarcinoma.

	**Univariate analysis**			**Multivariate analysis**			
	**pN0** **(*n* = 253)**	**pN1 (*n* = 40)**	***p*-value**	**Regression coefficient**	**Odds ratio**	**95% CI**	***p*-value**
Male sex	51 (20.2%)	10 (25%)	0.6232				
Age<45	92 (36.4%)	23 (57.5%)	0.0178	1.17840	3.2492	1.4531–7.2655	**0.0041**
Hyperthyroidism	38 (15%)	6 (15%)	0.8142				
Autoimmune thyroiditis	160 (63.2%)	15 (37.5%)	0.0036	0.84531	0.3488	0.1751–0.6948	**0.0408**
Nodule size ≥6 mm	119 (47%)	31 (77.5%)	0.0006	−0.93025	3.8786	1.7742–8.4791	**0.0476**
Thyroid weight	31.4 ± 32.4	36.7 ± 25.7	0.2381				
Histotype			0.0005				
PTC	164 (64.8%)	18 (45%)		1.000	1.000	Reference	
FV-PTC	56 (22.1%)	7 (17.5%)		0.26498	1.3034	0.4762–3.5678	0.6060
Tall cell carcinoma	33 (13%)	15 (37.5%)		1.24695	3.4797	1.3980–8.6610	**0.0074**
Extrathyroidal extension	14 (5.5%)	9 (22.5%)	0.0006	1.29253	3.6420	1.2302–10.7826	**0.0196**
Multicentric carcinoma	80 (31.6%)	17 (42.5%)	0.2388				
Angioinvasive carcinoma	10 (4%)	9 (22.5%)	<0.0001	1.25005	3.4905	1.1117–10.9595	**0.0322**

*PTC, papillary thyroid carcinoma; FV-PTC, follicular variant of PTC. Bold values indicates statistically significant*.

Multivariate analysis revealed age <45 years (36.4% in pN0 group vs. 57.5% in pN1 group; OR 3.249; *p* = 0.0041), nodule size ≥ 6 mm (47% in pN0 group vs. 77.5% in pN1 group; OR 3.878; *p* = 0.0476), tall cell variant of PTC (13% in pN0 group vs. 37.5% in pN1 group; OR 3.479; *p* = 0.0074), extrathyroidal extension (5.5% in pN0 group vs. 22.5% in pN1 group; OR 3.642; *p* = 0.0196), and angioinvasive carcinoma (4% in pN0 group vs. 22.5% in pN1 group; OR 3.49; *p* = 0.0322) as independent risk factors for lymph node metastasis. Conversely, autoimmune thyroiditis was found as a protective factor for LN metastasis (63.2% in pN0 group vs. 37.5% in pN1 group; OR 0.348; *p* = 0.0408).

**Figure 1:**
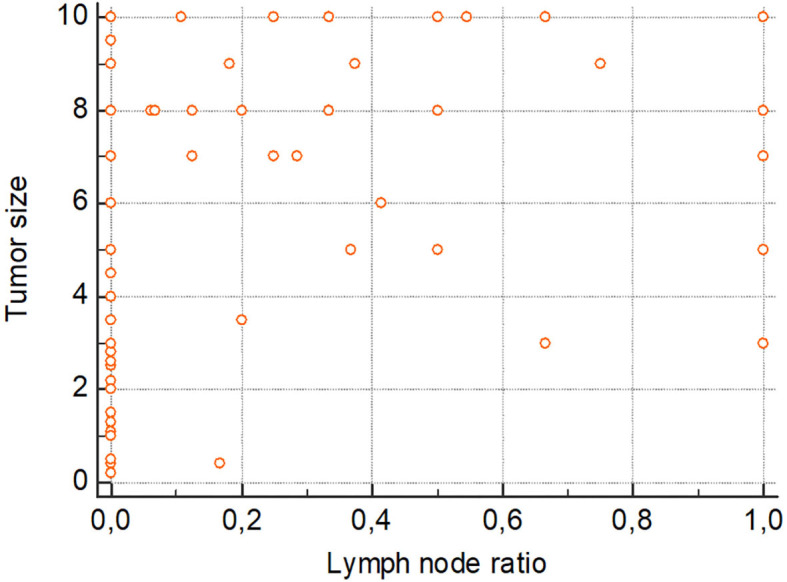
Scatter diagram reporting correlation between tumor size and lymph node ratio. Correlation coefficient = 0.2409; CI = 0.1134–0.3606; *p*-value = 0.0003.

As reported in Figure 1, a further analysis demonstrated a positive correlation between the tumor size and the lymph node ratio, with a correlation coefficient of 0.2409 (CI 0.1134–0.3606; *p* = 0.0003).

## Discussion

Papillary thyroid microcarcinoma is considered a tumor with an indolent behavior and a low risk of recurrence; for these reasons, ATA 2015 guidelines included this entity in the low-risk category, not recommending post-operative RAI, and also suggesting active surveillance management as an alternative to surgery in patients without evidence of lymph node metastasis or local invasion.

Nevertheless, in a subset of patients, PTMC exhibits aggressive behavior, presenting with lymph node metastasis and, in some cases, with local recurrence. The incidence of node metastasis in PTMC varies widely in the literature, ranging from 29.5 to 65% (8–10, 12–15), and node metastasis is considered the main predictive factor for local recurrence (16, 17).

The aim of this study was to assess potential risk factors for lymph node metastasis to identify patients with PTMC in whom aggressive surgery could be justified.

In the present study, ~13% of patients had lymph node metastasis of the central compartment, and 2% of the lateral neck compartment. Interestingly, we observed three cases of “skip metastasis” in which only the lateral compartment was involved, without evidence of node metastasis in the central compartment.

According to other studies present in the literature (12, 13, 18–23), we found younger age, tumor size, tall cell variant of PTC, extrathyroidal extension, and angioinvasion as independent risk factors for lymph node metastases.

It is interesting to note that size of the tumor appears to be an independent predictive factor for lymph node metastasis in almost all the series reported in the literature. Specifically, in our study we found an incidence of lymph node metastasis of 6.3% in patients with tumors with size up to 5 mm, and of 20.6% in patients with larger tumors; we also reported a significant correlation between tumor size and lymph node ratio.

Wang et al. (24) reported that 5.75 mm at preoperative ultrasound is the best cut-off value to predict the risk of central lymph node metastasis. In the study of Gong et al. (25), including over 1,100 patients, a cut-off value of 8.5 mm was calculated based on receiver operating characteristics (ROC) curves: tumors with larger size demonstrated an aggressive behavior with a poorer prognosis, including, other than a higher incidence of lymph node metastasis, the presence of extrathyroidal extension, multifocality, and local recurrence. Xu et al. (23) reported in a large series of over 3,000 patients with PTMC that a tumor size larger than 7 mm is strongly associated with a higher incidence of lymph node metastasis, suggesting in these patients a careful preoperative examination of central and lateral compartment and an aggressive surgical approach.

Furthermore, in our series, ~10% of patients with cN0 stage had lymph node metastasis at final pathology; when considering only patients with tumors larger than 6 mm, the incidence of unexpected lymph node metastasis was nearly 18%.

As extensively reported in Table 4, in our series, the subgroup of patients with tumors with size up to 5 mm presented significantly with non-aggressive features: as already remarked, the incidence of lymph node metastasis was 6.3% in this group, whereas it was 20.7% in tumors larger than 5 mm. Furthermore, angioinvasion was present only in 2 (1.4%) cases and extrathyroidal extension only in 5 (3.5%) patients in the first group, while it was found in 17 (11.3%) and 18 (12%) cases in the larger group, respectively.

**Table 4:** Characteristic of tumors considering a cut-off value of 6 mm.

	**Tumors <6 mm** **(*n* = 143)**	**Tumors ≥6 mm** **(*n* = 150)**	***p***
Nodule size (mm)	3.1 ± 1.5	8.3 ± 1.5	*p* < 0.001
Histotype			*p* < 0.001
PTC	105 (73.4%)	77 (51.3%)	
FV-PTC	28 (19.6%)	35 (23.3%)	
Tall cell carcinoma	10 (7%)	38 (25.3%)	
Extrathyroidal extension	5 (3.5%)	18 (12%)	0.0128
Multicentric carcinoma	32 (22.4%)	65 (43.3%)	*p* < 0.001
Angioinvasive carcinoma	2 (1.4%)	17 (11.3%)	0.001
Lymph node yield	3.4 ± 3.5	5.2 ± 5.6	0.001
Lymph node metastasis	9 (6.3%)	31 (20.7%)	*p* < 0.001
Number of involved LN per patient^*^	1.9 ± 2	2.7 ± 2.6	0.41
Lymph node ratio^*^	0.5 ± 0.3	0.5 ± 0.3	0.85

* *Calculated on 9 and 31 patients with metastatic lymph nodes in the first and second group, respectively*.

It is important to note that, among all the predictive factors of lymph node metastasis, the only feature preoperatively assessable is the size of the tumor; conversely, the other features, including extrathyroidal extension, tall cell variant of PTC, and angioinvasion, are pathological findings that are significant in follow-up but are not helpful for surgeons at the operating table.

Given all these considerations, it is reasonable to assume that the cut-off value of 10 mm, traditionally used as a discriminant for the definition of microcarcinoma, is too high to discriminate tumors with favorable pathological features from those with potential aggressive behavior that could require a more aggressive surgical approach.

As already stated, an active surveillance has been purposed for PTMC as an alternative to surgery (7). However, recent evidences deriving from the works of Choi et al. (26) and Oh et al. (27) suggested that the indication for active surveillance should be carefully weighted and applied for selected patients. In fact, the authors reported that some low-risk PTMC can progress significantly during the delayed intervention period for active surveillance, and that patients who underwent delayed surgical intervention had more aggressive disease and unfavorable oncologic outcome than those who underwent immediate surgery.

In our series, the diagnosis of PTMC was incidental in about 40% of the patients; in this case, even if the incidence of lymph node metastasis and of other pathologic aggressive features is uncommon, a risk stratification following ATA guidelines is recommended to identify potential tumors with medium or high risk of recurrence.

On the contrary, diagnosis of PTMC was made preoperatively in nearly 60% of the patients. In this case, an accurate preoperative US evaluation of cervical lymph nodes should always be performed, especially in tumors with size larger than 6 mm. Furthermore, intraoperative exploration of the VI level should be accurate, considering that US has low accuracy for node metastasis of the central compartment; a recent meta-analysis of Zhao et al., which included 19 articles and over 4,000 patients with PTC, reported a poor sensitivity of US in detecting metastases of the central compartment (pooled sensitivity of 33%, range 10–57%) with an incidence of lymph node metastases of 48% (28). Xue et al. reported similar results, with a sensitivity of US for diagnosis of metastatic lymph nodes of the central compartment of 22–55% (29).

A therapeutic neck dissection is always indicated in case of clinically involved lymph nodes. On the other hand, in case of clinically uninvolved lymph nodes, a prophylactic lymph node dissection of the central compartment is not indicated, also considering ATA guidelines.

Furthermore, it should be underlined that the incidence of lymph node metastasis depends from the tumor histotype. In our series, we found the tall cell carcinoma as an independent risk factor for lymph node metastasis: more than one third of patients with metastatic lymph nodes had a tall cell carcinoma. On the other hand, a follicular variant of PTC seems to be a less aggressive variant, with a lower incidence of lymph node metastasis, even if this difference was not significant in our series.

Another interesting finding is that we identified autoimmune thyroiditis as a protective factor for lymph node metastasis. In our series, the incidence of lymph node metastasis was 8.5% in patients with autoimmune thyroiditis and 21% in patients without autoimmune thyroiditis. This finding is discordant with other studies that identified autoimmune thyroiditis being associated with the aggressive behavior of PTC (30–32), including a higher incidence of lymph node metastasis. Indeed, some studies indicated that a different antibody status is associated with a distinct risk of development of PTC and of its prognosis: Paparodis demonstrated that high levels of TPOAb protect against DTC development in autoimmune thyroiditis (33), and Wen demonstrated that TPOAb are associated with a lower incidence of lymph node metastases (30).

This study has several limitations. First, this is a unicentric, retrospective study. Then, not all the patients underwent a complete CLND; thus, it is possible that the real incidence of lymph node metastasis is underestimated. Patients with autoimmune thyroiditis were not differentiated based on the different antibody status but were all included in the same category, and this could invalidate our finding of autoimmune thyroiditis as a protective factor for lymph node metastasis. In addition, a separate analysis was not performed to evaluate predictive factors for central or lateral lymph node metastasis. Finally, this study demonstrates a not negligible incidence of lymph node metastasis in PTMC, but, in this work, we did not evaluate if this finding has a significant prognostic value: further studies are required to specifically develop this issue.

## Conclusion

Despite the fact that PTMC is considered an indolent tumor with an excellent prognosis, the incidence of lymph node metastasis is high, even in patients with clinically uninvolved lymph nodes. The size of the tumor represents the most important predictive factor for lymph node metastasis, with tumors larger than 6 mm being at a higher risk for lymph node metastasis. Further studies could question the definition of PTMC to redefine a new cut-off value in order to better discriminate tumors with aggressive behavior, which could require aggressive surgery, from indolent tumors.

## Data Availability Statement

The datasets generated for this study are available on request to the corresponding author.

## Ethics Statement

The studies involving human participants were reviewed and approved by Comitato Etico Indipendente AOU Cagliari, Cagliari (Italy). The patients/participants provided their written informed consent to participate in this study.

## Author Contributions

FM, FB, ML, and PC contributed to the conception and design of the study. GC and FC organized the database and acquired the data. FM and EE performed the statistical analysis. FM wrote the first draft of the manuscript. FB, ML, EE, and PC critically revised the manuscript. All authors contributed to manuscript revision, read, and approved the submitted version.

## Conflict of Interest

The authors declare that the research was conducted in the absence of any commercial or financial relationships that could be construed as a potential conflict of interest.
